# Enhancing Title V Workforce Capacity to Address Complex Challenges: Impact of the National Maternal and Child Health Workforce Development Center

**DOI:** 10.1007/s10995-022-03430-5

**Published:** 2022-05-25

**Authors:** Alexandria M. Coffey, Laura Powis, Amy Mullenix, Vanessa Rivero, Shara Evans, Hiba Fatima, W. Oscar Fleming, Kristen Hassmiller Lich, Stephen Orton, Dorothy Cilenti, Lewis Margolis

**Affiliations:** 1 Department of Maternal and Child Health, University of North Carolina – Chapel Hill, 135 Dauer Drive, Chapel Hill, North Carolina 27599 United States; 2 The Association of Maternal and Child Health Programs, 1825 K Street Suite 250, Washington, DC 20006-1202 United States; 3 Kidzu Children’s Museum, 201 South Estes Drive, Chapel Hill, North Carolina 27514 United States; 4 Public Health Leadership Program, University of North Carolina – Chapel Hill, Box 7469, Chapel Hill, North Carolina 27599 United States; 5 Department of Health Policy and Management, University of North Carolina – Chapel Hill, 135 Dauer Drive, Chapel Hill, North Carolina 27599 United States; 6 North Carolina Institute for Public Health, University of North Carolina – Chapel Hill, 135 Dauer Drive, Chapel Hill, North Carolina 27599 United States

**Keywords:** Maternal and child health, Workforce, Leadership, Program evaluation, Transformation

## Abstract

**Introduction:**

The National Maternal and Child Health Workforce Development Center provides training, coaching, and consultation to Title V programs. The flagship experience is the Cohort program, a 6-8-month leadership development program where Title V programs convene a multisector team to address a pre-selected state/jurisdictional challenge related to health systems transformation. The overall objective of this paper is to demonstrate the impact of skills developed via the Cohort program on state/jurisdictional capacities to address complex challenges.

**Methods:**

Qualitative, post-Cohort evaluation data were analyzed using inductive and deductive coding and the “Sort and Sift, Think and Shift” method. Themes and supporting text were summarized using episode profiles for each team and subsequently organized using the EvaluLEAD methodology for identifying and documenting impact.

**Results:**

Teams brought an array of challenges related to health systems transformation and 94% of teams reported achieving progress on their challenge six-months after the Cohort program. Teams described how the Cohort program improved workforce skills in strategic thinking, systems thinking, adaptive leadership, and communication. Teams also reported the Cohort program contributed to stronger partnerships, improved sustainability of their project, produced mindset shifts, and increased confidence. The Cohort program has also led to improved population health outcomes.

**Discussion:**

Through working with the Center, Title V leaders and their teams achieved episodic, developmental, and transformative results through application of Center tools and skills to complex challenges. Investment in the MCH workforce through skill development is critical for achieving transformative results and solving “wicked” public health problems.

## Significance

### What is already known on this subject?

The Cohort program from the National Maternal and Child Health Workforce Center has been previously described (Margolis et al., 2017; Umble et al., 2022), along with episodic findings from evaluation data (Umble et al., 2022).

### What does this study add?

This study adds evidence that structured skill development for MCH professionals can have developmental and transformative workforce impacts, and advance population health outcomes.

## Introduction

Since 2013, the National Maternal and Child Health (MCH) Workforce Development Center (the Center), has provided training, coaching, and consultation to Title V programs. The Center’s mission is to enhance the capacity of Title V leaders and staff to engage in and lead health transformation efforts (Fleming et al., 2019). Title V leaders can include Title V Directors who administer MCH Block Grant funding as well as their program staff. As defined by the Center, health systems transformation includes five domains: (1) shifting the emphasis of healthcare from disease management to prevention and population health management, while improving access to affordable health care; (2) developing an interprofessional/interdisciplinary approach to health care; (3) integrating primary care, specialty care, and public health; (4) developing evidence-based, efficient health systems; and, (5) driving partnerships across sectors to optimize the wellbeing of MCH populations (Fleming et al., 2019; Margolis et al., 2017).

The Center provides training and technical assistance focused on three core areas: change management and adaptive leadership, evidence-based decision-making and implementation, and systems integration. Change management and adaptive leadership focuses on people and process dimensions of change, including team development and partnership sustainability. Evidence-based decision-making and implementation focuses on identifying and using rigorous methods to plan, promote, and sustain the implementation of effective programs, policies, and services. Systems integration addresses how systems interact with one another to shape and deliver public health and clinical services. Content on family partnership and health equity is integrated into each core area.

As described by Umble et al. (2022), the Center offers tailored trainings and technical assistance to meet the needs of each Title V program. The flagship experience is the Cohort program, a 6-8-month leadership development program where Title V convenes a multisector team to address a pre-selected state/jurisdictional challenge – generally a *wicked problem* – related to health systems transformation (Kreuter et al., 2004; *Wicked Problems*, n.d.). The Cohort program features intensive skill-based learning (i.e., multi-day Learning Institute) followed by individualized coaching to provide feedback, advice, referrals for Center experts, and assistance with measuring and tracking progress. Tools included in the Cohort program are briefly described in the Appendix and in Umble et al. (2022).

Evaluation of the Center’s trainings was guided by the EvaluLEAD methodology (Grove et al., 2005). EvaluLEAD focuses on identifying measurable results that flow from leadership and workforce development programs. The methodology suggests three distinct categories for identifying and documenting impact: episodic, developmental, and transformative.

1. *Episodic* results are defined as the “cause-and-effect” variety,” where an intervention takes place and results can be measured afterwards (Grove et al., 2005). Examples of such results could be learning a new skill or submitting a grant proposal.
2. *Developmental* results occur over a period of time at varying paces due to changes in external (e.g., reporting structure, funding availability) and internal factors (e.g., capacity, motivation) associated with advancing change. Developmental results are “sequences of steps taken by an individual, team, organization, or community that reach toward and may actually achieve some challenging outcomes” (Grove et al., 2005). Examples may include redesigning an existing program or implementing a new organizational strategic plan.
3. *Transformative* results are often unexpected findings that emerge when individuals or organizations take their work to new domains. Transformative results are “fundamental shifts in individual, organizational, or community values and perspectives that seed the emergence of fundamental shifts in behavior or performance” (Grove et al., 2005). Examples could include major changes in individual perspective or a new organizational mission.

Previous publications have described the Cohort program (Margolis et al., 2017; Umble et al., 2022), as well as presented episodic results (Umble et al., 2022). This study builds on these articles by describing the developmental and transformative outcomes reported by Title V teams. The overall objective of this paper is to demonstrate the impact of skills developed via the Cohort program on state/jurisdictional capacities to address complex challenges. We describe the skills and capacities Title V teams report from their Center experience, as well as how they applied these to their team’s “wicked” challenge.

## Methods

### Data sources and analysis

Our analysis is based on Cohort data from celebration webinars, as well as six-month and three-year interviews with program participants. The celebration webinar is a team presentation at the end of the Cohort program highlighting team accomplishments, Center tools they used, and skills they gained. Qualitative data from the celebration webinar were categorized using inductive coding methods, with codes emerging from individual responses.

A Center evaluator conducted structured interviews with state team lead(s) approximately six months, as well as three years, after the conclusion of the Cohort program. Prior to the scheduled interview, the Center evaluator sent the interview guide to team lead(s) for review and encouraged them to solicit input from team members. The primary focus of the interview was to gain insight on how the Center advanced their MCH population health goals through their team project. The interview guide included listing, multiple choice, and open-ended questions. The interviews lasted approximately one hour and were recorded and transcribed. Between 2014 and 2021, 51 six-month interviews (51/53 = 96% of teams) were completed and analyzed using ATLAS.ti. Evaluators coded the transcripts using a combination of deductive and inductive coding (*UNC Odum Institute for Research in Social Science*, n.d.). Deductive codes were developed based on objectives of the Cohort program. Inductive codes were developed in response to emerging themes from the interviews. The “Sort and Sift, Think and Shift” approach developed by ResearchTalk, Inc was used throughout the coding process (Maietta et al., 2019). To summarize findings, evaluators created an episode profile for each interview to describe the identified themes and supporting text, including data from three-year interviews when available (28/35 = 80% of teams).

## Results

**Fig. 1:**
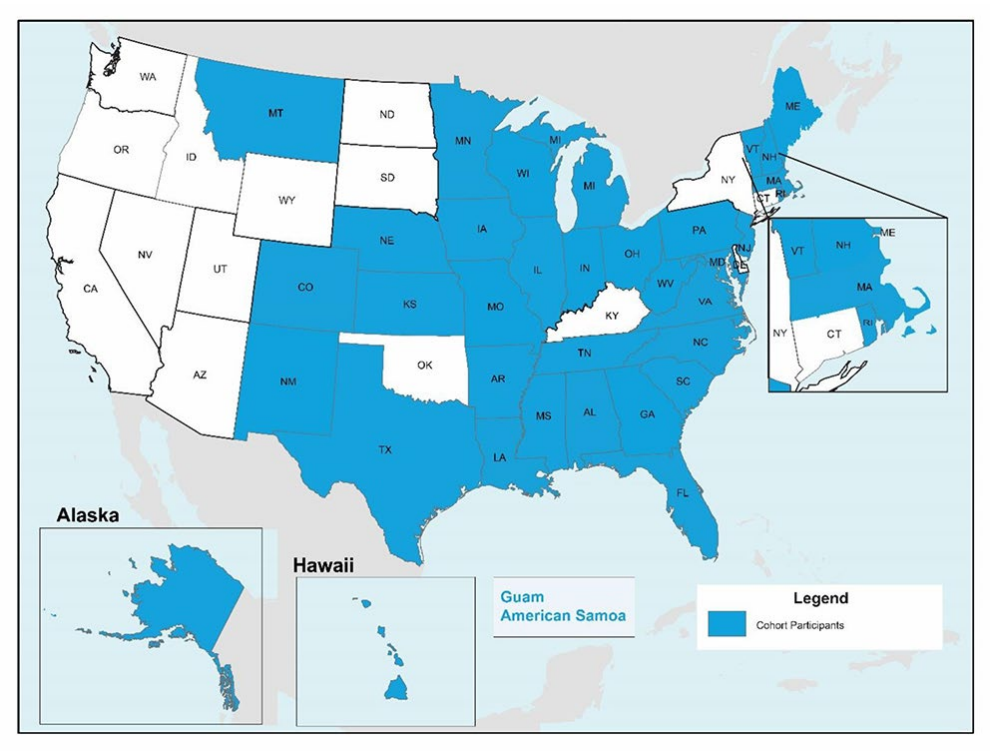
Map of state team participation in the Cohort program, 2014–2020 (n = 53)^1,^ ^2^. ^1^The Center has connected with nearly all non-Cohort participating states and offered/hosted other tailored programming to meet state needs, including customized technical assistance support and 3-day, in-state skills institutes. ^2^States/jurisdictions can participate in the Cohort program more than once. Oftentimes teams are addressing different complex challenges than in previous Cohorts and are comprised of different individuals; therefore, our sample size was determined by counting each time a state/jurisdiction participated in the Cohort program.

As shown in Table 1; Fig. 1, state teams from all four regions (Northeast, Midwest, West, and South) of the United States participated in the Cohort program between 2014 and 2020, with over 40% of teams from the South region. Additionally, 20 of the 53 teams represented states where greater than 30% of the state’s total population live in rural areas. State teams included an average of six different agency partners.

**Table 1:** Descriptive data for state teams participating in the Cohort program, 2014–2020 (n = 53)^1^

	Number of teams	Percentage of teams
U.S. Region		
Northeast	7	13.2
Midwest	14	26.4
West	9	17.0
South	23	43.4
Percent of total state population that lives in rural areas		
Less than 10%	8	15.1
Between 10-19.9%	8	15.1
Between 20-29.9%	17	32.1
Greater than 30%	20	37.7
Average number of agencies represented across all teams^2^	6.2	

^1^ Some states completed the Cohort Program more than once; therefore, they are counted more than once in the above table

^2^ One state did not provide information on the number of agencies represented in their team

### Team accomplishments

State teams brought challenges across a wide range of domains, including efforts to strengthen health systems, service delivery, and partnerships (Table 2). For example, eight states addressed ways to increase alignment and collaboration among different services providing developmental screening, such as early intervention, home visiting, and primary care. Across these domains, the majority of teams reported reaching their challenge goals to a high degree (n = 26) or somewhat reaching their challenge goals (n = 22) by their six-month interview. In the following sections, we describe how teams achieved progress on their challenge through reaching a variety of developmental and transformative results.

**Table 2:** Overview of the focus of team projects, 2014–2020 (n = 53)

Focus	Number of teams	Example project
Enhance service delivery	9	Create a toolkit for telehealth implementation to increase system capacity to address the needs of families with children and youth with special healthcare needs
Strengthen and streamline screening services	8	Increase support of evidence-based developmental screening in child care programs across the state
Strengthen health system	7	Improve sustainability of MCH systems of care initiatives in local communities by broadening their knowledge-base of sustainability and identifying a framework to support their work
Enhance stakeholder engagement	6	Engage young adults and partners to increase young adult utilization of health care services
Enhance care coordination	6	Improve the systems of care for children and youth with special healthcare needs by facilitating cross-agency collaboration and developing a children’s health services work group
Integrate primary care, specialty care, and public health	4	Promote integration of behavioral health into primary care for children, youth, adolescents, and young adults by developing a toolkit with the Department of Education and increasing visibility through an expanded and strengthened partnership base
Build and strengthen partnerships	4	Engage private and public partners in process improvement and work with rural tribal populations to incorporate their experiences
Enhance alignment/consolidation of work	4	Build a comprehensive equity plan and operationalize new practice throughout all MCH programs
Transition to population health services	3	Develop one year action plan to realign priority needs and performance measures of MCH programs and funding in the state with MCH 3.0 Transformation
Improve youth transition to adulthood	2	Strengthen partnerships with stakeholders and create a shared understanding of the children and youth with special healthcare needs transitioning system by convening a workgroup

### Developmental results

#### Enhanced workforce capacity

From their experience with the Center, participants described improved workforce skills primarily in four areas: (1) strategic thinking, (2) systems thinking, (3) adaptive leadership, and (4) communication.

As noted in Table 3, more than one-third of teams reported enhanced strategic thinking skills at the end of the Cohort program. Participants reported elements of strategic thinking, such as: identifying elements of complex problems; creating smaller, concrete steps to address problems; and identification and utilization of relevant tools to proceed. One participant working to develop a comprehensive system of care through strengthened partnerships described strategic thinking as “starting with a big overview, a big problem, a big area of focus and peeling back the onion and really getting to the root of what we wanted” (Team 1).

**Table 3:** Knowledge and skills reported by Title V teams during the Celebration Webinar at the end of the Cohort Program, 2014–2020 (n = 53)^1^

Category	Number of teams (%)
Enhanced ability to engage key players using Center tools^2^	32 (60)
Enhanced knowledge of Center tools	29 (55)
Enhanced adaptive leadership/teamwork	26 (49)
Increased understanding of needs for solving MCH challenges	21 (40)
Enhanced strategic thinking	20 (38)
Enhanced knowledge of MCH topics, other than skills	11 (21)
Enhanced understanding of issues around health transformation	10 (19)
Applied evidence to inform Title V work	4 (8)

^1^ Teams can provide more than one accomplishment/knowledge/skill gained through the cohort training program with the Center; therefore, the numbers above do not sum to the total sample size. If a team noted any accomplishment/knowledge/skill more than once, it was only counted once.

^2^ Examples of Center tools include system support maps, 30/30, and impact matrix.

Participants described enhanced systems thinking throughout their Center experience. As noted in Table 3, over 50% of teams reported having an increased understanding of Center tools to address complex challenges (see Appendix A for descriptions of Center Curriculum Tools). Additionally, 60% of teams reported an improved ability to engage partners with Center tools, such as system support maps. The increased knowledge and application of systems thinking allowed teams to better tackle complex MCH challenges. One participant working to improve stakeholder buy-in and support for developmental screenings elaborated: “…within our own division [we] realiz[ed] that we need to be more integrated. [L]ooking at outcome[s] and not just output[s], and not just [talking about] individual programs, like developmental screening, but how that applies to general child health” (Team 2). In this example, understanding the interconnectedness of individual programs, such as home visiting and early intervention, and how that leads to improving child health demonstrates use of systems thinking skills. Center tools, such as process maps and the 5 R’s, were helpful to teams for identifying their role in their organization and within the larger public health system, as well as for understanding barriers to advancing system changes (see Appendix A for tool descriptions).

Nearly half of teams reported enhanced adaptive leadership skills, including mutual learning (Schwarz, 2013) (Table 3). Participants expressed how Center tools helped improve their ability to build effective teams based on personality preferences, skills, and expertise. For example, one participant, whose project focused on developing a comprehensive outreach plan for early and periodic screening, diagnosis, and treatment, described their commitment to team building through mutual learning with the Center.

“…a lot of what we needed their help with was…organizing as a team and figuring out how to manage the work. It wasn’t really…the logistics of the projects that we ultimately needed their help with. It was…how do we come together as a team? Because a really interesting group of people [are] in this work group that all come from very different backgrounds and have different personalities and different workstyles.” (Team 3)

Adaptive leadership skills also enhanced work beyond teams’ projects as participants were able to apply skills in work extending from their challenge. One participant, whose team’s project focused on developing and integrating regional networks of access and quality to improve care for children and youth with special healthcare needs (CYSHCN), used change management skills when working with stakeholders to improve their pediatric palliative care program.

“It’s exciting to be able to go to things and…know how to tackle this challenge. [W]e have tools for this. We’ve done this before and we’re going to follow the process. We may not have the solution, but we…have the tools we need to explore it. We’ve been able to model that change management-type behavior…with [our partners].” (Team 4)

Learning and applying Center skills to another project not only improved their workforce capacity, but also their partners’ through sharing professional development opportunities and bringing their partners along through the learning process.

Participants gained techniques to strengthen communication, recognizing such techniques as methods to help overcome complex challenges. Examples of improved communication skills included finding common language to build collective understanding and developing agreed upon, politically acceptable language. One participant, working to engage key stakeholders to realign MCH program priorities and needs with the MCH 3.0 Transformation (MCH Navigator, 2017), described how Center tools informed their communication strategy to advocate for a new MCH position to their department’s leadership team. “[The] way he presented it helped [the leadership team]…understand how we were moving from where we were in the past, where we were presently, and how we needed to move for the future. They got it, they came on board, and [were] willing to accept it and do what was necessary to make the transformation a reality” (Team 5).

Collectively, participants described how the Cohort program provided opportunities to apply these four skills in other areas of their work, beyond the challenges they addressed in the cohort. For example, one participant explained how their team applied system support mapping on an information technology project separate from their team challenge. “We’re building a new electronic system and…it will house several of our programs. [W]e used [system support mapping]…and our IT people were so impressed with how we were able to quickly organize the information into user stories that they are going to turn around and replicate it [into] other things that they do” (Team 6).

#### Stronger partnerships

Title V promotes and improves the health and well-being of mothers, children, including CYSHCN, and their families through the creation of partnerships with governmental and non-governmental agencies and programs. Many stakeholders—early childhood, public instruction, Medicaid, nutrition—influence a broad array of determinants of the well-being of MCH populations. Title V programs can leverage the resources of the MCH Services Block Grant Program to enhance their impact through multi-sector partnerships. Teams described how Center tools and skills advance these types of partnerships.

Enhanced systems thinking contributed to stronger partnerships. Systems mapping tools, for example, brought together organizations and agencies which had not previously worked together. One team reported how system support maps were an effective tool to engage partners, such as Medicaid, private insurers, and policy and advocacy groups, on how to build a comprehensive development framework for early intervention screening:

“We had conversations with the leads with a lot of these programs when we were doing our mapping activities around who was making referrals, who was doing screening services and such…at the stakeholder meetings. When we did those system support maps, I think that also helped to illuminate everybody’s role in this—that we hoped to eventually build an effective, integrate[d] early childhood development system.” (Team 7)

Working with the Center also increased teams’ diversity of partners. For example, the Cohort program supported teams in developing deeper connections with Medicaid by including Medicaid leaders on the team. A number of teams reported systems tools created opportunities to work more closely with state Medicaid programs. Specifically, such tools provided a common language to explore strategies to improve care coordination for families of CYSHCN. In one state, for example, Medicaid, the Title V CYSHCN program, and managed care organizations came together to define and implement shared plans of care for families across agencies. This common language reduced duplication across systems and increased efficiency in care coordination services across the state.

“[In the Cohort program] we have the opportunity to further enhance our collaborative skills…[W]e have not historically had the same collaboration with our Medicaid agency as we have in that past couple of years…this opportunity gave us a reason to pull in someone from Medicaid, and because there was this formalized application process…and travel to a two-day meeting, they had to designate someone and commit to a certain level of time and energy to be part of it. I think we got a higher level of buy in because of [the Cohort program].” (Team 8)

Having the Center itself as a partner increased the credibility of teams when approaching potential collaborators. For example, one participant described a challenging relationship with Medicaid on the issue of healthcare homes. Engagement with the Center legitimized their team’s status as a partner and allowed them to push back on Medicaid’s funding approach which failed to acknowledge the different service needs between children and adults with chronic conditions. “[It] was really helpful to have an outside entity help us with that because, internally, I think we would not have been as successful because there is a lot of history between the two sections” (Team 9).

#### Sustainability

Several workforce development skills increased their opportunities to sustain projects and programs. Participants reported that sharing Center tools with stakeholders led to increased buy-in, ultimately facilitating sustainability of their work. One team focused on reducing adverse asthmatic experiences for children by coordinating a system of care through community partnerships. Center skills helped engagement, resulting in collaborative grant applications to continue their efforts. The team also reflected on how achieving sustainability of the project was a key indicator of success, even when the initial team members were no longer engaged in the work. “[I]t doesn’t need your skills at the time because you’ve kind of passed the baton and that’s an important thing to do in the state for sustainability and for having the community take the lead on projects” (Team 10).

### Transformative results

Teams reported transformative results, that is, “fundamental shifts in individual, organizational, or community values and perspectives that seed the emergence of fundamental shifts in behavior or performance” (Grove et al., 2005). One team, working to integrate evidence-based behavioral health treatment guidelines into primary care settings, shared their “experience with the Center has impacted everything [they] do.” The team further elaborated how evidence-based decision-making influenced their work:

“You guys didn’t give us a magic pill or a magic answer. You gave us tools to help us critically think through this project…[and] applied those tools to help us critically think and tease-out other complex problems that we had…you made us sit down and…think outside the box. [N]ot only did you give us tool[s], but…you gave us the practice space to apply them. I’ve had implementation science as part of a college program, but being able to actually have didactic [learning] with real world problem[s] just helped it come to life.” (Team 6)

Another team, also promoting behavioral health integration into primary care settings, shared how evidence-based decision-making and implementation science is something they “think about almost daily when looking at different programs…[their] department is initiating” (Team 11). Specifically, the team shared how their perspectives have shifted to focus on the installation of adequate and appropriate training, support, and communication of new initiatives, all key elements of implementation.

Another transformative result was increased confidence. One team described how increased confidence led to enhanced work with their community partners. “[S]he feels more comfortable [with] sharing the data, sending the data out, and getting feedback…I think it’s the level of confidence with themselves and…they’re more comfortable working together, supporting, participating, and providing more information [about] their initiative” (Team 12). Another Title V team, who worked on improving systems of care for CYSHCN through cross-agency collaborations, described how working with the Center increased their confidence to have a more direct conversation with their director about what can reasonably be achieved with existing funding sources. “From the cohort we were able to have confidence to stand up to the director and say, ‘MCH Title V money is not enough for all the things you want’” (Team 13).

### Impact on population health

Collectively, developmental and transformative results were associated with improved population health. One team’s project focused on creating a state certification program for their Community Health Workers (CHWs), so their services could be reimbursed by Medicaid and other insurers (Team 14). Three years after completing the Cohort program, the state reported 232 CHWs had been certified and were working within multiple community agencies and programs. Families working with CHWs experienced tremendous improvements in population health measures, such as a nearly 50% decline in missed school days among children with asthma and increased use of clinician-advised asthma action plans from 20 to 80% (Alexander-Scott & Dunklee, 2018).

Another example is how teams applied Center tools and skills to subsequent public health challenges. One team used Center tools and skills to adapt and advance strategies in response to the Zika outbreak (Team 13). The team used systems mapping tools to create a roadmap to help families navigate systems and access services. Using quality improvement skills and tools from the Cohort program allowed the team to continuously review what was working and not working, adjust the roadmap to be more effective in connecting families with services, and obtain and integrate community feedback. The team’s work on responding to the Zika outbreak led to a greater focus on incorporating the voices of parents, more specifically among families with CYSHCN. Change management tools, including appreciative inquiry, were used to guide implementation of a family-to-family health information center to support greater family participation in their Title V program. Additionally, greater family engagement led to an agreement between Title V and the public works transportation program to register CYSHCN with a transportation system to assist with medical appointments and household errands.

## Discussion

Title V leaders and their teams achieved episodic, developmental, and transformative results through application of Center tools and skills to complex challenges. The Center’s three core areas and tools provided an approach and the workforce capacity to address this complexity. This intentional approach provided teams with resources—space, time, coaching, tools, skills—to identify their aspirations, recognize how and where they are experiencing difficulties, and work together towards creating change. Further, this approach increased confidence by providing a mental “safety net” for individuals and teams to try hard things they might otherwise not attempt. The Center’s approach can also be a catalyst for eliciting deeper and more systemic workforce development, as the Cohort program enhanced work beyond their chosen state/jurisdictional projects.

Our analyses have limitations. Given time and energy investments by teams and the Center, there may be social desirability in interview responses; however, interviewees were asked to provide concrete justifications/explanations to validate their responses. There was also no control group to allow for measurement of impact relative to states/jurisdictions not engaged in the Cohort program; however, many respondents noted participation in the Cohort program was more effective than other technical assistance programs. Teams may have also had exposure to other leadership development/capacity building programs or other technical assistance during their follow-up period of the Cohort program. Individual exposure to other programs was not collected and could be a potential limitation of our analysis.

Collecting long-term follow-up data enabled us to identify how the Center’s capacity-building approach inspired positive changes in mindset, organizational structure, confidence, as well as population health outcomes. Future evaluation of workforce development programs should include long-term follow-up to illuminate improvements in population health, as well as unanticipated impacts that may be overlooked amidst more pressing, urgent needs for Title V programs. Additionally, state MCH leadership structures vary considerably across states. Future evaluation should seek to measure and contextualize findings within each state’s MCH leadership structure to better understand the influence of state leadership structures in addressing complex challenges. Evaluation tools should also be designed to collect more data on how workforce development programs influence diversity and equity outcomes.

The Center has demonstrated that investment in the MCH workforce is critical for solving “wicked” public health problems, such as health equity, access to care, and attention to social determinants of health (e.g., housing, education, economics). The Center continually reviews the training and technical assistance needs of Title V leaders and adapts its’ approach, curriculum, skills, and tools accordingly. Through this iterative process and partnership, the Center seeks to empower Title V leaders with the skills and tools necessary to address “wicked” public health problems in their state.

## Data Availability

Not applicable.

## Appendix A. Explanation of Center Curriculum Tools

**Table:**

**Tools to Develop the Team**	
**Core Conversations**	This tool guides groups through a series of questions to explore multiple aspects of their work together, including strengths, dissent, and commitment to the work.
**Conversational Sweet Spot**	This tool helps individuals balance candor and curiosity in conversations to promote positive relationships with colleagues.
**Coat of Arms**	This creative activity relies on individual and team strengths to produce a visual representation of a team’s transformation challenge.
**Network Map**	This tool allows individuals to visually represent the strength and density of stakeholder relationships that are currently or could be leveraged to support team efforts.
**System Support Map**	A deep-dive mapping exercise to depict an individual’s responsibilities, needs, experience with available resources, and wishes. It can be used to describe consumer needs, team success, or define and strengthen an MCH system/initiative.
**Tools to Understand the Challenge**	
**5Rs**	A conversation guide to describe the system individuals work in by depicting diverse perspectives about success, roles, resources to support change, and rules and relationships that must be understood or changed to improve outcomes.
**Individual Challenge Statements**	A structured exercise that prompts teams to capture diverse perspectives on a complex challenge, allowing team members to understand stakeholder priorities and vocabulary.
**Group Challenge Statement**	This tool consolidates multiple individual challenge statements into a consensus document.
**Aim Statement**	Often building on a Group Challenge Statement, an aim statement provides a concise description of project goals and vision, clearly stating time frame and perspective.
**Causal Loop Diagram**	Causal Loop Diagrams are used to elicit and integrate mental models, examine root causes of challenges, and identify key leverage points for action.
**Tools to Consider Action Steps**	
**Implementation Staging**	A framework to examine the “life course” of a complex effort and identify action steps related to each implementation stage.
**Synthesize the Evidence**	This tool can be used to organize and summarize key information/findings from your search for “what works.”
**Key Driver Diagram**	A visual summary of the overall strategy that illustrates pathways of change and priority focus areas.
**Implementation Supports Checklist**	A checklist to prompt consideration of the organizational, leadership, and competency supports.
**Tools to Document and Communicate**	
**Team Roster**	Team rosters are used to clarify roles in complex projects. Implementation team rosters are used to document roles of individuals on implementation teams.
**Communication Protocol**	A tool to document agreements with stakeholders with whom the team needs to share information and from whom the team needs information.
**30/30**	A tool to track the progress and learning of the team.
